# Co-fibrillogenesis of Wild-type and D76N β_2_-Microglobulin: THE CRUCIAL ROLE OF FIBRILLAR SEEDS*

**DOI:** 10.1074/jbc.M116.720573

**Published:** 2016-02-26

**Authors:** Antonino Natalello, P. Patrizia Mangione, Sofia Giorgetti, Riccardo Porcari, Loredana Marchese, Irene Zorzoli, Annalisa Relini, Diletta Ami, Giulia Faravelli, Maurizia Valli, Monica Stoppini, Silvia M. Doglia, Vittorio Bellotti, Sara Raimondi

**Affiliations:** From the ‡ Department of Biotechnology and Biosciences, University of Milano-Bicocca, 20126 Milan, Italy,; the § Consorzio Nazionale Interuniversitario per le Scienze Fisiche della Materia (CNISM), UdR Milano-Bicocca, 20126 Milan, Italy,; the ¶ Wolfson Drug Discovery Unit, Centre for Amyloidosis and Acute Phase Proteins, Division of Medicine, University College London, London NW3 2PF, United Kingdom,; the Departments of ‖ Molecular Medicine, Institute of Biochemistry, and; the ** Internal Medicine and Therapeutics, University of Pavia, 27100 Pavia, Italy, and; the ‡‡ Department of Physics, University of Genoa, 16146 Genoa, Italy

**Keywords:** amyloid, fibril, Fourier transform IR (FTIR), protein aggregation, protein misfolding, β2-microglobulin

## Abstract

The amyloidogenic variant of β_2_-microglobulin, D76N, can readily convert into genuine fibrils under physiological conditions and primes *in vitro* the fibrillogenesis of the wild-type β_2_-microglobulin. By Fourier transformed infrared spectroscopy, we have demonstrated that the amyloid transformation of wild-type β_2_-microglobulin can be induced by the variant only after its complete fibrillar conversion. Our current findings are consistent with preliminary data in which we have shown a seeding effect of fibrils formed from D76N or the natural truncated form of β_2_-microglobulin lacking the first six N-terminal residues. Interestingly, the hybrid wild-type/variant fibrillar material acquired a thermodynamic stability similar to that of homogenous D76N β_2_-microglobulin fibrils and significantly higher than the wild-type homogeneous fibrils prepared at neutral pH in the presence of 20% trifluoroethanol. These results suggest that the surface of D76N β_2_-microglobulin fibrils can favor the transition of the wild-type protein into an *amyloid* conformation leading to a rapid integration into fibrils. The chaperone crystallin, which is a mild modulator of the lag phase of the variant fibrillogenesis, potently inhibits fibril elongation of the wild-type even once it is absorbed on D76N β_2_-microglobulin fibrils.

## Introduction

The conversion of globular native proteins into amyloid fibrils represents the crucial pathogenic event of systemic amyloidoses and its molecular mechanism has been extensively studied *in vitro* (1). For prototypic globular amyloidogenic proteins, such as lysozyme (2), transthyretin (TTR) (3), and wild-type β_2_-microgobulin (WT β_2_m)^2^ (4), the fibrillogenesis *in vitro* is primed by non-physiological conditions including high temperature, prolonged incubation at acidic pH, or addition of organic solvent. The natural amyloidogenic variant of β_2_m (D76N) has provided the first example in which the fibrillogenesis of a globular full-length amyloidogenic protein can be achieved in a physiological environment. Thermodynamic destabilization caused by the single point mutation (D76N) was shown to play an essential role in enhancing the amyloidogenic propensity of this globular protein (5). Furthermore, we have demonstrated that the energy required to misfold D76N β_2_m is compatible with the energy provided by shear forces present in living human organs (6). The discovery of biocompatible conditions of fibrillogenesis is very informative and useful to recapitulate events occurring *in vivo* as well as for testing putative inhibitors of the process for further pharmacological exploitation. Physiological methods of fibrillogenesis are also an essential tool for studying the phenomenon of copolymerization of the amyloidogenic variant and the wild-type counterpart as occurs in some autosomic dominant forms of familial amyloidoses. The mechanism of copolymerization of putative normal and pathological conformers in protein misfolding diseases is still elusive. The structure and conformation of propagon (7) are not yet determined both in prion diseases (8) and in specific types of amyloidosis, such as familial transthyretin amyloidosis, in which amyloid deposits formed by the variant seed the fibrillar conversion of the wild-type protein (9). In other types of autosomic dominant forms of systemic amyloidosis, such as those caused by lysozyme variants in patients heterozygous for the mutation, only the variant polymerizes into fibrils, whereas the wild-type counterpart escapes from the amyloid conversion (2). It is plausible that copolymerization may occur when the wild-type isoform is intrinsically amyloidogenic (*i.e.* TTR) and not in cases in which the wild-type never forms, *per se*, amyloid fibrils *in vivo* (*i.e.* lysozyme). Amyloidosis caused by β_2_m is quite peculiar because, despite its intrinsic amyloidogenic propensity (10), the wild-type is not deposited in amyloid fibrils of heterozygous carriers of the D76N mutation. This finding is particularly surprising because, *in vitro*, this variant can efficiently prime the fibrillogenesis of wild-type β_2_m (6). We have already showed that the truncated form of β_2_m lacking the first N-terminal residues (ΔN6 β_2_m) can trigger oligomerization of the wild-type protein (11). More recently, a *prion-like* property was attributed to ΔN6 β_2_m based on its capacity to prime the amyloid conversion of the wild-type through a monomer/monomer interaction (12). This mechanism is under discussion and our data suggest that both ΔN6 β_2_m and the full-length D76N variant can induce the amyloid conversion of wild-type β_2_m only after their own fibrillar transformation, thus suggesting that copolymerization is caused by a mechanism of elongation over heterologous seeds rather than by a *prion-like* activity. Because D76N β_2_m is much more potent and efficient than ΔN6 β_2_m in priming the wild-type fibrillogenesis, we have further analyzed the structural events as they occur in the WT during its copolymerization with the variant β_2_m and described the prevalent mechanism.

## Experimental Procedures

Production of recombinant β_2_m isoform (WT β_2_m and D76N β_2_m) were expressed and purified as previously described (5). [^13^C]WT β_2_m was also produced using Spectra 9 minimal medium containing 98% ^13^C (Cambridge Isotope Laboratories, Inc.).

### 

#### 

##### Fibrillogenesis Time Course Procedure

Fibrillogenesis was carried out in glass vials stirred at 750 rpm at 37 °C using 50 μm β_2_m isoforms in PBS, pH 7.4. Aggregation was monitored by thioflavin T (ThT) emission at 480 nm after excitation at 445 nm (13). β_2_m, which remained soluble during aggregation, was monitored by native gel electrophoresis. The soluble fraction was separated by centrifugation at 20,817 × *g* for 10 min before loading onto 1% agarose gel and bands were quantified with Quantity One software (Bio-Rad). Fibrillogenesis experiments were also conducted in the presence of 10 μm α-crystallin (Sigma).

##### Electron Microscopy

Formvar-coated copper electron microscopy (EM) grids were placed coated side down onto each sample and incubated for 2 min before blotting with filter paper to remove excess solvent and staining with 2% (w/v) uranyl acetate for 2 min. After further blotting and drying in air, transmission electron microscope (CM120) images were obtained at 80 keV.

##### Cross-seeding Fibrillogenesis

Samples of WT β_2_m, 100 μl at 40 μm in PBS, pH 7.4, containing 10 μm ThT (13) were incubated at 37 °C in Costar 96-well black-wall plates sealed with clear sealing film in the absence or presence of D76N β_2_m fibrils (1.7 μm) or S52P TTR fibrils (1.4 μm) (14). Bottom fluorescence was recorded at 8-min intervals (BMG LABTECH FLUOstar Omega). Relative intensities of ThT emission were monitored in three replicate test and control wells for 10 h.

##### Fourier Transform Infrared Spectroscopy (FTIR)

The protein conformational changes occurring in the time course of β_2_m fibrillogenesis were monitored by FTIR measurements in attenuated total reflection. For these analyses, 2 μl of the protein samples were deposed on the single reflection diamond crystal of the attenuated total reflection device (Quest, Specac, USA) and dried at room temperature to obtain a protein hydrated film (15, 16). FTIR spectra of the hydrated films were collected by the Varian 670-IR spectrometer (Varian Australia Pty Ltd., Mulgrave VIC, Australia) under the following conditions: 2 cm^−1^ resolution, scan speed of 25 kHz, 1000 scan coadditions, triangular apodization, and a nitrogen-cooled Mercury Cadmium Telluride detector. Spectra were smoothed using the Savitsky-Golay method before the second derivative analysis, both performed with the Resolutions-Pro software (Varian Australia Pty Ltd., Mulgrave VIC, Australia).

##### Determination of Fibril Stability

Fibrillar material for equilibrium denaturation experiments was prepared using 100 μm protein. D76N β_2_m fibrils or the equimolar mixture of D76N/WT were prepared under stirring conditions at 750 rpm in PBS buffer, pH 7.4, at 37 °C. WT β_2_m fibril formation was carried out in 50 mm phosphate buffer containing 100 mm NaCl, pH 7.4, in the presence of 20% (v/v) trifluoroethanol (TFE) at 37 °C and pre-formed WT β_2_m seeds at 20 μg/ml. D76N β_2_m fibrils were also prepared in the presence of 20% (v/v) TFE for comparison. After 7 days incubation, fibrillar aggregates were quantified by assessment of the monomer left in the supernatant considering that the extinction coefficient (1 mg/ml) is 1.691 for both WT and variant D76N β2m. Fibrils (0.5 mg/ml) in PBS, pH 7.4, were incubated with increasing concentrations of guanidine hydrochloride (GdnHCl) from 0 to 7 m. Samples were mixed by vortexing and incubated at room temperature for 24 h as this time was experimentally verified to allow the samples to reach equilibrium. To separate non-aggregated from aggregated protein, samples were centrifuged in a Beckman Optima TLX ultracentrifuge at 125,000 × *g* for 60 min. The monomer concentration in the supernatant was quantified by measuring the absorbance at 280 nm as previously described (17). The fraction of soluble monomeric β_2_m over the total concentration was plotted with denaturant concentration.

The electrophoretic analysis of β_2_m soluble samples under native conditions was conducted after removal of denaturant. Gel bands were quantified with Quantity One software (Bio-Rad).

##### Determination of Thermodynamic Stability Parameters

The equilibrium unfolding curves of β_2_m fibrils were analyzed using a linear polymerization model (17–19) [*F_i_* − 1] + [*M*] [*F_i_*], in which [*M*] and [*F_i_*] represent the concentration of monomers and fibrillar aggregates of size *i*, respectively, with the equilibrium constant *K* = *c*_0_[*F_i_*]/[*F_i_* − 1][*M*], where *c*_0_ is the standard concentration 1 mol liter^−1^. Based on this model the fraction of monomeric β_2_m over the total protein concentration, [*M*]/[*M_T_*], can be expressed as Equation 1.

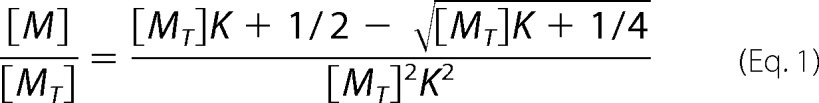
 The equilibrium constant *K* can also be expressed as *K* = exp(–Δ*G*_el_/*RT*), in which Δ*G*_el_ is the free energy of elongation, *R* is the gas constant, and *T* the absolute temperature. In the presence of chemical denaturants, *i.e.* GdnHCl, Δ*G*_el_ is linearly dependent on the concentration of denaturant, [*D*], according to Δ*G*_el_ = m[*D*] + Δ*G*_el_^0^, where m is a cooperativity coefficient and Δ*G*_el_^0^ is the free energy of elongation in the absence of denaturants (17). The experimental data of the equilibrium unfolding of WT and D76N fibrils were fitted to Equation 1 to obtain the main thermodynamic parameters using KaleidaGraph 4.0 (Synergy Software, Reading, PA). Values of midpoint denaturant concentration, [*D*]_50%_ were also calculated. All measurements are reported as mean ± S.D. of three independent experiments.

##### Equilibrium Denaturation Experiments of Monomeric β_2_m

GdnHCl equilibrium denaturation of both monomeric WT and D76N variant β_2_m were performed at 20 °C in PBS, pH 7.4, as previously described (6).

##### Fibril Elongation

Samples of WT or D76N β_2_m, 50 μm, were incubated in PBS, pH 7.4, at 37 °C under stirring in the presence of 50 μm D76N β_2_m fibrils grown in the absence or presence of 10 μm α-crystallin. Aggregation of β_2_m was monitored by quantifying the soluble fractions of WT and D76N β_2_m by 8–18% polyacrylamide gradient gels (ExcelGel, GE Healthcare) and by ThT fluorescence emission at 480 nm after excitation at 445 nm (13).

##### Atomic Force Microscopy (AFM)

For AFM inspection, 40-μl sample aliquots were centrifuged at 1700 × *g* for 5 min using an Eppendorf 5417R centrifuge. The pellet was suspended in an equal volume of water, and a 10-μl aliquot was deposited on freshly cleaved mica and dried under mild vacuum. Tapping mode AFM images were acquired in air using a Dimension 3100 Scanning Probe Microscope equipped with a “G” scanning head (maximum scan size 100 μm) and driven by a Nanoscope IIIa controller, and a Multimode Scanning Probe Microscope equipped with “*E”* scanning head (maximum scan size 10 μm), driven by a Nanoscope V controller (Digital Instruments, Bruker). Single beam uncoated silicon cantilevers (type OMCL-AC160TS, Olympus) were used. The drive frequency varied between 280 and 330 kHz, the scan rate was between 0.4 and 0.7 Hz. Height and width of imaged objects were measured from the corresponding cross-section profiles in topographic AFM images. Widths at half-height were measured to correct tip size effects (20) and standard errors are reported. The object volume V was calculated from the equation,

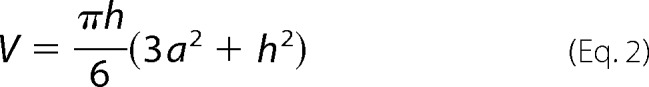
 where *h* is the imaged object height and *a* is its half-corrected width (20).

## Results

### 

#### 

##### Interactions between D76N β_2_m and WT β_2_m during Fibrillogenesis under Physiological Conditions

**FIGURE 1.**
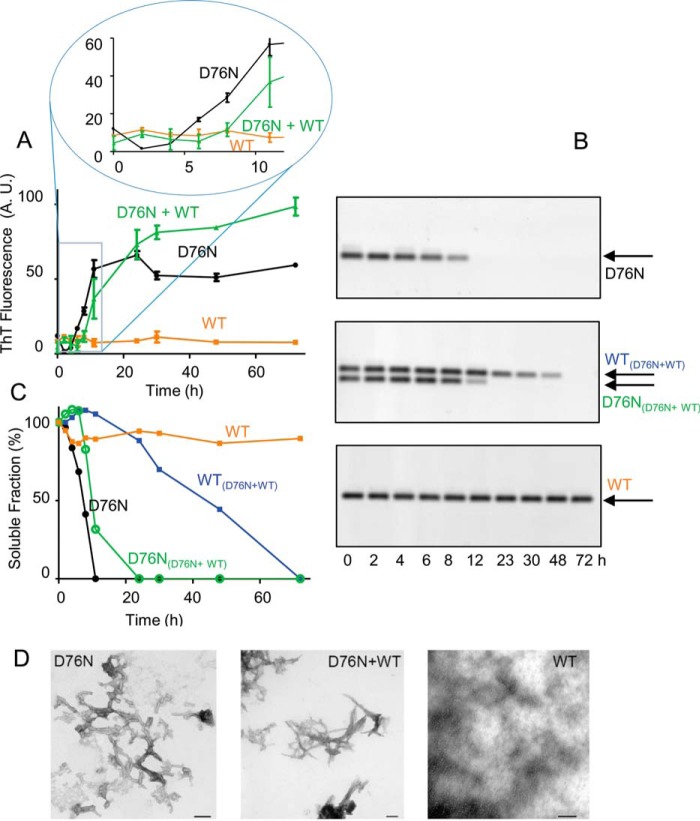
**Fibrillogenesis of D76N and WT β_2_m.** *A*, the time course of aggregation of D76N β_2_m, WT β_2_m, equimolar mixture of D76N β_2_m and WT β_2_m under stirring conditions, at 37 °C, was monitored by ThT fluorescence emission with excitation and emission wavelengths at 445 and 480 nm, respectively. *Inset*, expanded view of the ThT signal between 0 and 12 h. *B*, agarose gel electrophoresis analysis of supernatants from fibrillogenesis samples as described above. The *arrows* show the electrophoretic mobility of each isoform. *C*, density of agarose gel bands were measured and plotted as soluble fractions with time. Values shown in *A* and *C* are mean ± S.D. (*error bars*) from three independent experiments. *D*, negatively stained transmission electron microscopy (*scale bar*, 100 nm) showing that only WT β_2_m alone does not form fibrils under physiological conditions and in the absence of D76N β_2_m seeds.

**FIGURE 2.**
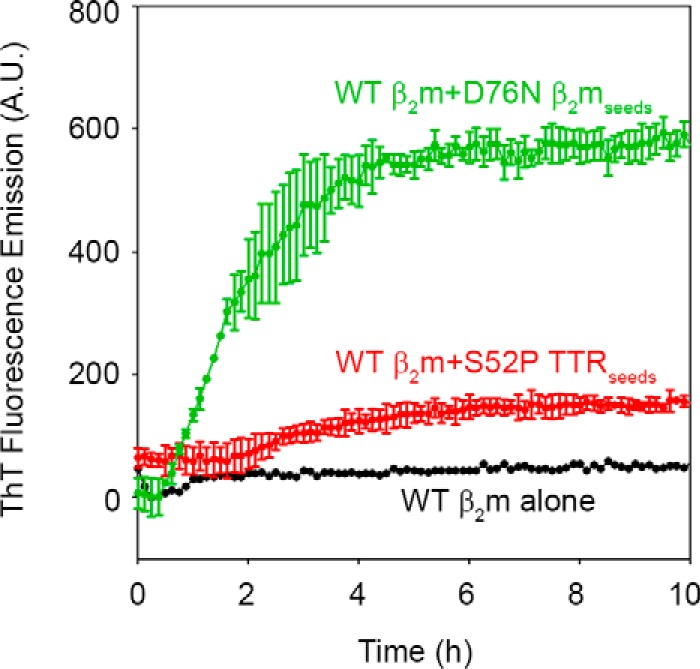
**Cross-seeding fibrillogenesis of WT β_2_m.** Time course of aggregation of WT β_2_m (40 μm) in the absence or presence of D76N β_2_m seeds (1.7 μm) or S52P TTR seeds (1.4 μm) as described under “Experimental Procedures.” Relative intensities of ThT emission, after subtraction of the corresponding seeds fluorescence, were plotted with time. Mean ± S.D. (*error bars*) from three independent experiments. *A.U*., arbitrary units.

Fibrillogenesis and solubility of β_2_m were, respectively, monitored by measuring the increase in the ThT fluorescence (13) (Fig. 1*A*) and by quantifying the soluble fractions of WT and D76N, which can be readily differentiated in native 1% agarose gel electrophoresis (Fig. 1, *B* and *C*) based on their different electrophoretic mobilities. As we already showed (6), D76N β2m rapidly converted into fibrils after a lag-phase of ∼6 h, whereas WT β_2_m did not form fibrils under the same conditions and time frame (Fig. 1*D*). Because patients carrying the D76N mutation are expected to express both WT and variant, we further investigated the aggregation kinetics of an equimolar mixture of the two species. In this case, the lag time of the D76N variant was slightly, but consistently, prolonged (Fig. 1), suggesting that its fibrillar conversion was negatively affected by the interaction with the wild-type. This experiment confirmed (6) that the variant primed the fibrillar conversion of the wild-type, which aggregated after a lag time of ∼24 h (Fig. 1). WT β_2_m did not aggregate in the presence of seeds from different sequence fibrils, such as S52P TTR fibrils, in which ThT emission was monitored in microplate wells (see “Experimental Procedures”) (14) (Fig. 2), suggesting that the WT β_2_m conformational conversion required a highly specific fibrillar template.

##### β_2_m Aggregation Monitored by Isotope-edited FTIR Spectroscopy

To characterize the structural changes occurring in each β_2_m isoform during aggregation, we carried out FTIR spectroscopy analysis in which the formation of intermolecular β-sheets in protein supramolecular assemblies can be detected by analyzing the Amide I band, mainly associated with the CO stretching vibrations of the peptide bonds in the 1700–1600 cm^−1^ spectral region (21–23).

Determination of the individual contribution to the spectral changes, in a mixture of WT and variant β_2_m, is experimentally challenging, but is practicable when one of the two species is labeled with ^13^C. Indeed, the replacement of ^12^C with ^13^C in WT β_2_m typically leads to a downshift of the Amide I band components of about 40–45 cm^−1^, enabling us to study the conformational properties of both labeled and unlabeled proteins (24).

**FIGURE 3.**
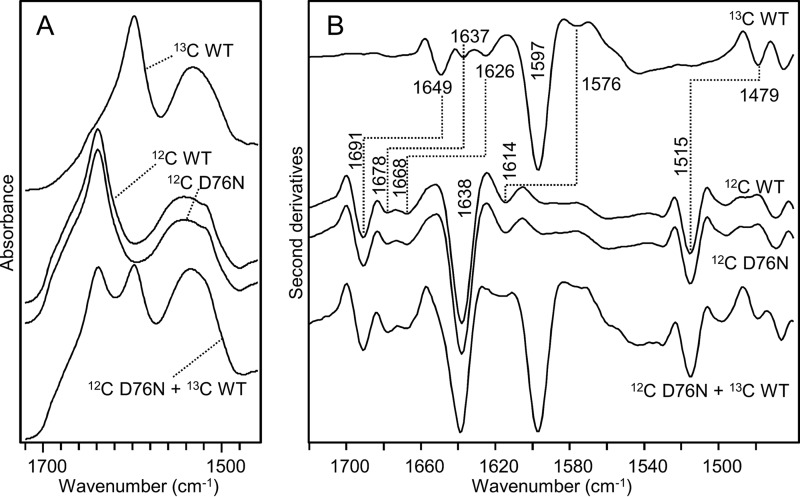
**FTIR spectra of unlabeled (^12^C) and isotopically labeled (^13^C) β_2_m.** *A*, absorption spectra of native [^12^C]WT β_2_m, [^13^C]WT β_2_m, [^12^C]D76N, and an equimolar mixture of [^13^C]WT and [^12^C]D76N. *B*, second derivatives of absorption spectra shown in *A*. Peak positions of the main components are indicated. The absence of the peak at 1691 cm^−1^ in the ^13^C protein confirms that isotopic labeling was successfully achieved.

This effect is clearly shown in Fig. 3, where the absorption spectra of unlabeled (^12^C) and isotopically labeled (^13^C) WT β_2_m are reported together with the spectra of the [^12^C]D76N variant and an equimolar mixture of [^13^C]WT and [^12^C]D76N β_2_m (Fig. 3*A*).

To resolve the Amide I band into its overlapping components, we performed the second derivative analyses in which the minima correspond to the maxima in the original spectra. Accordingly to their peak positions, these components can be assigned to the protein secondary structures.

The second derivative spectra of [^12^C]WT and D76N proteins (Fig. 3*B*) displayed two components at ∼1691 and ∼1638 cm^−1^ due to the native antiparallel β-sheet structures. In addition, two peaks at ∼1678 and ∼1668 cm^−1^ were assigned to turn structures, whereas an additional peak observed at ∼1614.5 cm^−1^ was assigned to β-sheets or amino acid side chains. These results are in agreement with FTIR characterizations of WT β_2_m previously reported (25–27).

All the Amide I components and the tyrosine peak at ∼1515 cm^−1^ in the unlabeled and, at 1479 cm^−1^ in the labeled proteins, respectively (Fig. 3*B*), were downshifted of about 40 cm^−1^ in the ^13^C species as expected (28). In the 1700–1600 cm^−1^ region, where the Amide I band of the [^12^C] β_2_m occurred, only minor contributions of the isotopically labeled protein were observed. This allowed us to study the conformational transitions of the [^12^C]D76N variant that took place during its aggregation also in the presence of the ^13^C-labeled WT protein. Indeed, in the second derivative spectrum of the equimolar mixture of the two variants, the native β-sheet components (at ∼1691 and ∼1638 cm^−1^) of the [^12^C]D76N variant could be clearly discriminated from the main native β-sheet peak of the [^13^C]WT protein at ∼1597 cm^−1^ (Fig. 3*B*).

**FIGURE 4.**
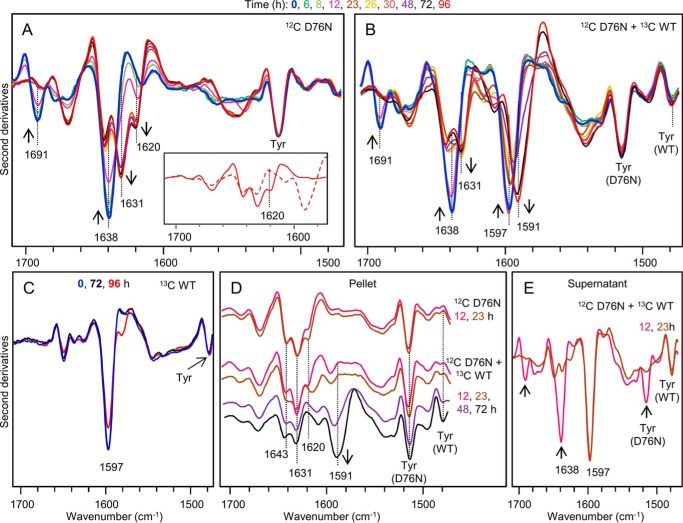
**Time course of β_2_m aggregation studied by isotope-edited FTIR spectroscopy.** *A*, second derivatives of absorption spectra of [^12^C]D76N variant β_2_m at 50 μm at different times of incubation at 37 °C (*blue*, time 0; *red*, time 96 h). *Inset*, expanded view of the second derivative spectra of the aggregates by [^12^C]D76N variant alone (*solid line*) and by [^12^C]D76N/[^13^C]WT β_2_m equimolar mixture (*dashed line*) after 96 h of incubation. *B*, second derivatives of absorption spectra of an equimolar mixture of [^12^C]D76N/[^13^C]WT β_2_m, both at 50 μm, at different times of incubation at 37 °C. *C*, second derivatives of absorption spectra of [^13^C]WT β_2_m at 50 μm at different times of incubation at 37 °C. *D*, second derivatives of absorption spectra of the pellet, and *E*, of the supernatant obtained by centrifugation of aliquots withdrawn from the same samples analyzed. Only spectra at selected incubation times are shown. The *arrows* point to the spectral changes occurring with time. Spectra are reported after normalization at the Tyr peak around 1515 cm^−1^ in D76N β_2_m (*A*, *B*, and *D*), at the Tyr peak of WT β_2_m (*C*), or at the native β-sheet peak at ∼1597 cm^−1^ in WT β_2_m (*E*).

Based on these results (Fig. 3), we monitored the aggregation of [^12^C]D76N β_2_m either alone (Fig. 4*A*) or in the mixture with the [^13^C]WT protein (Fig. 4*B*) by infrared spectroscopy. FTIR analysis of [^13^C]WT β_2_m alone was carried out under the same conditions as control (Fig. 4*C*).

The second derivative spectrum of native [^12^C]D76N (Figs. 3*B* and 4, *A* and *B*) was identical to that of native [^12^C]WT β_2_m (Fig. 3*B*), indicating that the mutation did not induce major changes in the protein secondary structures in agreement with both crystallographic and NMR data (5, 6). During the incubation of [^12^C]D76N β_2_m at 37 °C and under stirring conditions, several spectral changes took place including decrease of the ∼1691 and ∼1638 cm^−1^ peaks (due to the native intramolecular β-sheets) (see *arrows* in Fig. 4*A*). Also the peak at ∼1678 cm^−1^ (due to turn) decreased in intensity after 6–8 h of incubation. The ∼1614.5 cm^−1^ component of the native protein appeared to move up to ∼1620 cm^−1^ with increasing intensity. Furthermore, a new component at ∼1631 cm^−1^ was observed and assigned to the formation of intermolecular β-sheets together with the contribution at ∼1620 cm^−1^. This process occurred without the appearance of intense absorption components around 1695–1680 cm^−1^ implying that the formation of intermolecular β-sheets may follow a parallel orientation of the β-strands in the final aggregate (29, 30). In addition to these peaks, other components at ∼1670 and ∼1643 cm^−1^ were observed in the final aggregate although their assignment could not be unequivocally done. Indeed, they may be associated with turns, loops, or with a peculiar arrangement of the β-strands in the protein supramolecular assemblies (27, 31).

FTIR analysis of the equimolar mixture of [^12^C]D76N and [^13^C]WT β_2_m (Fig. 4*B*) showed that, at the beginning of the incubation, the second derivative spectrum of the mixture displayed the peak components of both native [^12^C]D76N variant and [^13^C]WT β_2_m. Native D76N β_2_m components in the mixture started to decrease in intensity after 8–12 h of incubation at 37 °C and the new peaks, due to protein aggregation, appeared in the second derivative spectra (see *arrows* in Fig. 4*B*). Also in the mixture, the WT protein unfolded and aggregated as indicated by the decrease in native β-sheet peak at ∼1597 cm^−1^ and the raising of the new component at ∼1591 cm^−1^, assigned to the formation of intermolecular β-sheets in the [^13^C]WT protein. Noteworthy, the aggregation of WT β_2_m started only after extensive aggregation of the variant, as highlighted by FTIR analyses of pellet and supernatant after centrifugation (Fig. 4, *D* and *E*).

Only the spectral pattern of D76N β_2_m aggregates could be observed in the second derivative spectra of the pellet obtained by centrifugation of the mixture after 12 and 23 h of incubation. In this case, the IR peaks assigned to [^13^C]WT appeared in the spectra of the pellet after 48 h incubation and their intensity increased at 72 h (Fig. 4*D*). On the contrary, WT β_2_m alone maintained its native secondary structures and its soluble state during 72 h incubation under the same conditions. In this case, detectable spectral changes appeared only after 96 h incubation (Fig. 4*C*). The supernatant of the mixture at 12 h displayed the peak components of both native [^12^C]D76N and [^13^C]WT proteins, whereas the spectrum of the mixture at 23 h (Fig. 4*E*) was very similar to that of the native [^13^C]WT protein alone (Fig. 3*B*). These results clearly indicated that after 23 h of incubation, most of the D76N variant in the mixture became insoluble and fibrillar, whereas the WT maintained its native soluble state (Fig. 4, *D* and *E*) until the complete conversion of the variant into fibrils (Fig. 4*B*).

**FIGURE 5.**
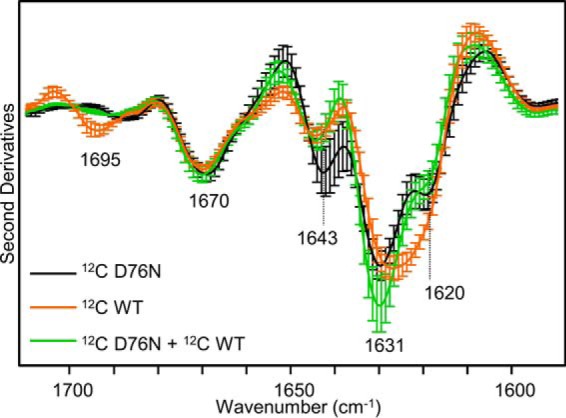
**Second derivative spectra of mature β_2_m fibrils.** Second derivative spectra of fibrils by D76N β_2_m alone or by an equimolar mixture of WT and D76N β_2_m formed after incubation at 37 °C under shear forces and, by WT β_2_m at neutral pH in the presence of 20% TFE. Isotopically unlabeled proteins were used. Mean ± S.D. (*Error bars*) of spectra from 3 independent fibril preparations are shown.

The final aggregate of the mixture [^12^C]D76N and [^13^C]WT proteins (Fig. 4*B*), obtained after 96 h of incubation, showed spectroscopic features overlapping those of the variant alone (peaks at ∼1670, ∼1643, and ∼1631 cm^−1^), except for a decreased intensity in the peak at ∼1620 cm^−1^ (Fig. 4*A*, *inset*) indicating that the aggregates of the two proteins may interact to some extent. This minor difference in the D76N IR response either in the mixture or alone could be due to the formation of a small amount of mixed β-sheets (*i.e.* with β-strand provided by the two species) and/or to the supramolecular packing of the fibrils. To explore the possible formation of polymorphic β_2_m fibrils, the second derivative spectra of the fibrils of the isotopically unlabeled proteins were performed (Fig. 5), and showed only minor differences in their fibril secondary structures or supramolecular packing. The second derivative spectrum of the fibrils obtained from an equimolar mixture of the two species under shear forces revealed analogous spectral components, with similar intensities, compared with those of the D76N β_2_m fibrils (main peaks at ∼1670, ∼1643, ∼1631, and ∼1620 cm^−1^). In this case, the ∼1631 cm^−1^ peak of the mixture was higher than that of the D76N alone; however, in consideration of their standard deviations, the two FTIR responses became partially overlapping (Fig. 5). As a control, we used homogeneous WT β_2_m fibrils obtained at neutral pH in the presence of 20% TFE as proposed by Yamaguchi *et al.* (32). The second derivative spectrum of these mature WT β_2_m fibrils revealed spectral components similar to those of D76N fibrils (Fig. 5). Noteworthy, these components were characterized by differences in their relative intensities, indicating that the two fibrils may be structurally different. In particular, the ∼1620 cm^−1^ component had a higher relative intensity in the WT β_2_m fibrils prepared in TFE.

##### Thermodynamic Analysis of β_2_m Fibrils

To determine how the structural differences observed for each fibril type may influence their stability, we titrated the different types of β_2_m fibrils with GdnHCl and then quantified the amount of soluble material released from the corresponding fibrillar species after 24 h incubation at different concentrations of denaturant.

**FIGURE 6.**
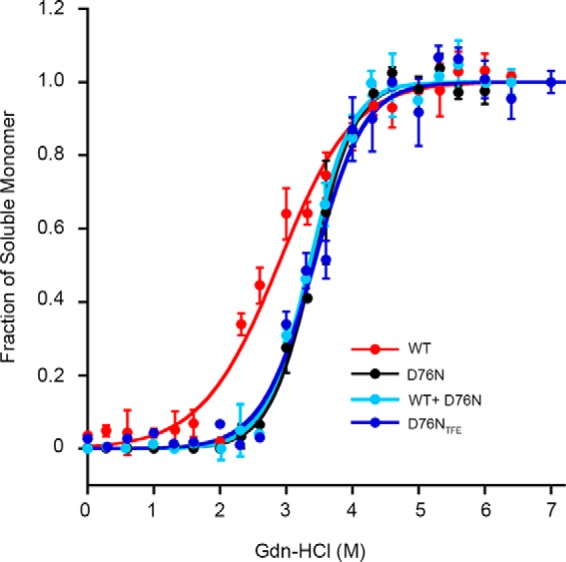
**Thermodynamic stability of *in vitro* fibrils.** The proportion of monomer released from β_2_m fibrils over the total protein concentration at increasing GdnHCl concentrations was analyzed with Equation 1 following the linear polymerization model as described under “Experimental Procedures.”

Fractions of each quantified soluble monomer over the corresponding total protein concentration were fitted with the linear polymerization model as described under “Experimental Procedures” (17). The results showed that regardless of the growth conditions used to prepare the D76N β_2_m fibrils, they were significantly more stable than those formed by WT β_2_m in the presence of 20% TFE (Fig. 6). The midpoint concentration of denaturant was 3.4 m for D76N β_2_m fibrils grown either in physiological conditions or in the presence of 20% TFE. This value dropped to 3.0 ± 0.28 m for WT β_2_m fibrils (Table 1). Interestingly, the hybrid WT/D76N β_2_m fibrils acquired the same thermodynamic stability as the homogenous D76N β_2_m fibrillar aggregate both yielding a difference in free energy of elongation (ΔΔ*G*_el_^0^) with WT β_2_m fibrils of approximately −3 kcal mol^−1^ (Table 1).

**TABLE 1:** **Thermodynamic parameters of GdnHCl induced unfolding of β_2_m fibrils and monomers** All values are mean ± S.D. of three independent experiments.

	Fibrils*^a^*		Monomers*^b^*	
	[*D*]_50%_	Δ*G*_el_^0^	[*D*]_50%_	Δ*G*_0_(H_2_O)
**WT*^c^***	3.0 ± 0.28	−9.3 ± 0.36	2.2 ± 0.2	6.2 ± 1.0
**D76N*^d^***	3.4 ± 0.15	−12.8 ± 0.35	1.6 ± 0.1	4.4 ± 1.1
**D76N_TFE_*^c^***	3.4 ± 0.20	−12.0 ± 0.64		
**Hybrid D76N/WT*^d^***	3.4 ± 0.14	−12.5 ± 0.41		

*^a^* Values of [*D*]_50%_ (*M*), midpoint concentration of GdnHCl and Δ*G*_el_^0^ (kcal mol^−1^), free energy of association in absence of denaturant were calculated following the linear polymerization model (17).

*^b^* [*D*]_50%_ (*M*) and Δ*G*_0_(H_2_O) (kcal mol^−1^), free energy of unfolding in absence of denaturant for equilibrium denaturation of monomeric WT and D76N β_2_m were determined using a two-state model previously described (44).

*^c^* Fibrils formed in the presence of 20% TFE (see “Experimental Procedures”).

*^d^* Fibrils formed in physiological conditions as described under “Experimental Procedures.”

**FIGURE 7.**
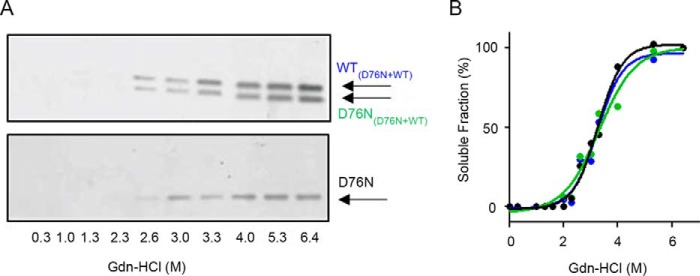
**WT and D76N β_2_m are simultaneously released from the hybrid fibrils.** *A*, agarose gel electrophoresis analysis of refolded soluble fractions of fibrils formed under shear forces by an equimolar mixture of WT/D76N β_2_m or D76N β_2_m alone (see “Experimental Procedures”) at different denaturant concentrations. *B*, soluble fraction measured by density of gel bands was plotted with denaturant concentration showing that the same amount of WT and D76N β_2_m was released from the mixed fibrils and that a similar quantity of soluble D76N β_2_m was generated during the disassembly of the corresponding homogenous fibrils.

To determine whether monomers of WT β_2_m and D76N β_2_m were equally released from hybrid fibrils during GdnHCl denaturation, the soluble fractions at different denaturant concentrations were refolded and analyzed by native gel electrophoresis. The results showed that an equal amount of both monomeric species was simultaneously released during the disassembly of fibrils (Fig. 7). Furthermore, both WT and D76N β_2_m fibrils were more stable than the corresponding globular monomeric precursors (Table 1), confirming that the aggregation pathway moves toward more stable structures (33).

##### Effect of α-Crystallin on Fibrillogenesis of D76N β_2_M and WT β_2_M

We reported (6) that the prototypic extracellular chaperone protein, α-crystallin, was an effective inhibitor of amyloid conversion of WT β_2_m seeded by D76N β_2_m aggregates and that high concentrations of α-crystallin were able to slow down the kinetics of fibrillar conversion of D76N β_2_m. Here we further investigated the interaction of α-crystallin with β_2_m in the aggregation pathway.

**FIGURE 8.**
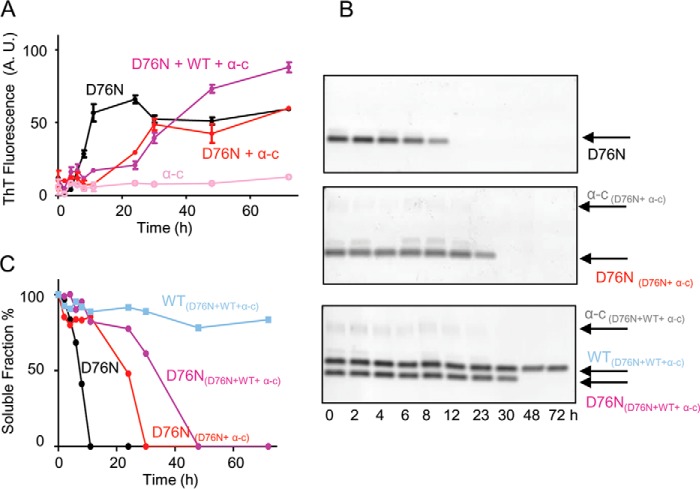
**Fibrillogenesis of D76N β_2_m in the presence of α-crystallin and WT β_2_m.** *A*, time course of aggregation of D76N β_2_m alone, D76N β_2_m in the presence of α-crystallin (α-*C*), an equimolar mixture of WT and D76N β_2_m in the presence of α-crystallin and, α-crystallin alone was monitored under stirring conditions by fluorescence emission of ThT. Protein concentrations were 50 μm for each β_2_m isoform and 10 μm for α-crystallin, respectively. Data are mean ± S.D. of three independent experiments. *A.U*., arbitrary units. *B*, agarose gel electrophoresis analysis of supernatants from one series of fibrillogenesis samples containing D76N β_2_m alone, D76N β_2_m in the presence of 10 μm α-crystallin, and equimolar mixture of WT and D76N β_2_m in the presence of 10 μm α-crystallin. *C*, soluble fraction quantified by density of gel bands and plotted with time.

At a 5:1 molar ratio of D76N β_2_m/α-crystallin, the lag phase of D76N β_2_m fibril formation extended from ∼6 to ∼23 h (Fig. 8) thus suggesting that the chaperone may interfere with the nucleation phase of D76N β_2_m fibrillogenesis. Co-presence of α-crystallin and WT β_2_m (Fig. 8) significantly affected the lag phase of D76N fibrillogenesis (from 8 to 30 h) and, under those conditions, WT β_2_m did not polymerize.

**FIGURE 9.**
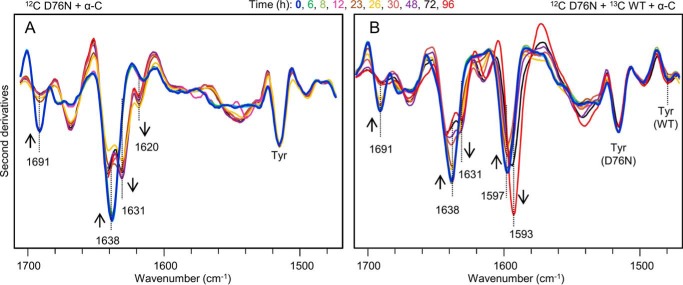
**Time course of β_2_m aggregation in the presence of α-crystallin studied by isotope-edited FTIR spectroscopy.** *A*, second derivatives of absorption spectra of 50 μm [^12^C]D76N in the presence of 10 μm α-crystallin collected at different times of incubation, 37 °C. *B*, second derivatives of absorption spectra of an equimolar mixture of the two β_2_m species in the presence of α-crystallin (50 μm [^12^C]D76, 50 μm [^13^C]WT, 10 μm α-crystallin) at different times of incubation, 37 °C.

The structural effects of α-crystallin were also analyzed by FTIR (Fig. 9). The conformational changes of β_2_m observed during the incubation at 37 °C were found to be similar in the presence and absence of the chaperone. However, their time courses were different, particularly with the equimolar mixture of the two β_2_m isoforms.

**FIGURE 10.**
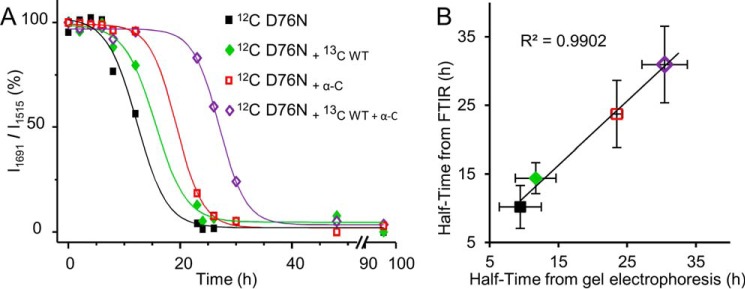
**Time course of D76N aggregation.** *A*, time course of the intensity of the ∼1691 cm^−1^ component of the D76N variant, due to the native β-sheet structures. The intensities at ∼1691 cm^−1^ were normalized at the tyrosine peak of the variant (at ∼1515 cm^−1^) and given as percentage variation. *B*, the aggregation half-time of D76N β_2_m under different conditions was obtained from the FTIR data and compared with that obtained from the electrophoretic analyses.

The delay in D76N β_2_m aggregation in the presence of the WT protein with and without α-crystallin could be better appreciated in Fig. 10*A*, where the time course of the ∼1691 cm^−1^ peak of the native D76N is reported for the different samples examined in this study. Indeed, the ∼1691 cm^−1^ component emerged as a specific marker for the native D76N β_2_m because it was strongly reduced in the final aggregates and it was absent in the [^13^C]WT protein.

Therefore, variations in the intensities of peaks at ∼1691 cm^−1^ can be monitored directly in the second derivative spectra after normalization at the tyrosine peak of the variant (at ∼1515 cm^−1^) to account for possible differences in the protein content. In Fig. 10*B*, the half-time of the process as determined from the intensity changes of the ∼1691 cm^−1^ peak of the native D76N (Fig. 10*A*) highly correlated to that obtained by native electrophoresis (Figs. 1*C* and 8*C*) for the different samples examined in this study.

We clearly observed that α-crystallin co-precipitated with D76N β_2_m fibrils (Fig. 8*B*) as the band of the soluble chaperone disappeared as soon as D76N β_2_m began to aggregate. It should be noted that α-crystallin incubated alone and in the same experimental conditions, did not convert into fibrillar aggregates (Fig. 8*A*). Interestingly, although α-crystallin co-precipitated with β_2_m fibrils, it was still able to inhibit the aggregation of WT β_2_m, suggesting that α-crystallin can prevent fibril elongation once it is bound to mature fibrillar aggregates.

**FIGURE 11.**
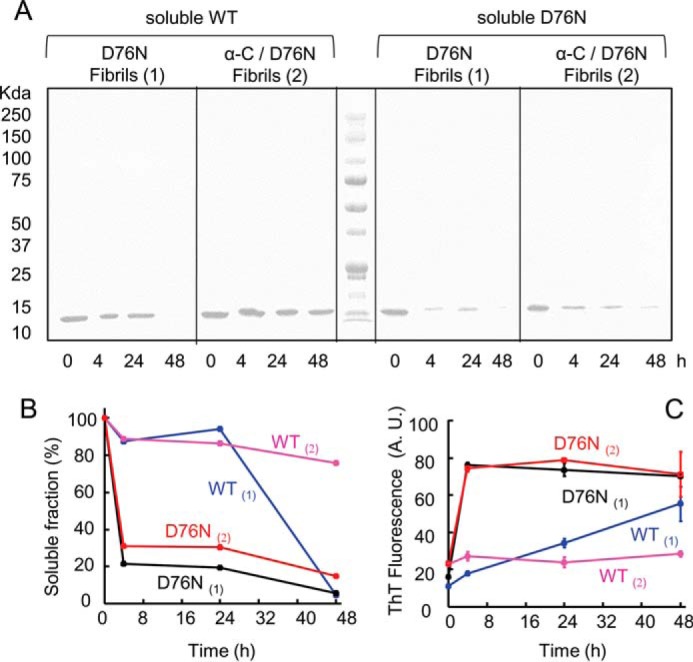
**Residual soluble WT β_2_m or D76N β_2_m during aggregation in the presence of pre-formed fibrils.** *A,* SDS-PAGE electrophoresis analysis of the soluble fraction of WT and D76N β_2_m at different times of incubation in PBS, 37 °C, under stirring conditions in the presence of D76N β_2_m fibrils alone (*1*) or in association with α-crystallin (*2*). *B,* density of gel bands in *A* were measured and plotted as soluble fractions with time. *C,* aggregation was monitored by ThT fluorescence emission with excitation and emission wavelengths at 445 and 480 nm, respectively.

To confirm this hypothesis in different conditions, WT or D76N β_2_m was incubated at 37 °C under stirring conditions in the presence of D76N β_2_m fibrils grown with or without α-crystallin. The analysis of the soluble fraction after 4, 24, and 48 h of incubation (Fig. 11) showed that when α-crystallin is associated with D76N β_2_m fibrils, WT β_2_m is protected from the seeding effect of D76N β_2_m fibrils; on the contrary the effect of the chaperone on the elongation phase is minimal (Fig. 11).

##### AFM Images of Crystallin-Fibrils Interaction

**FIGURE 12.**
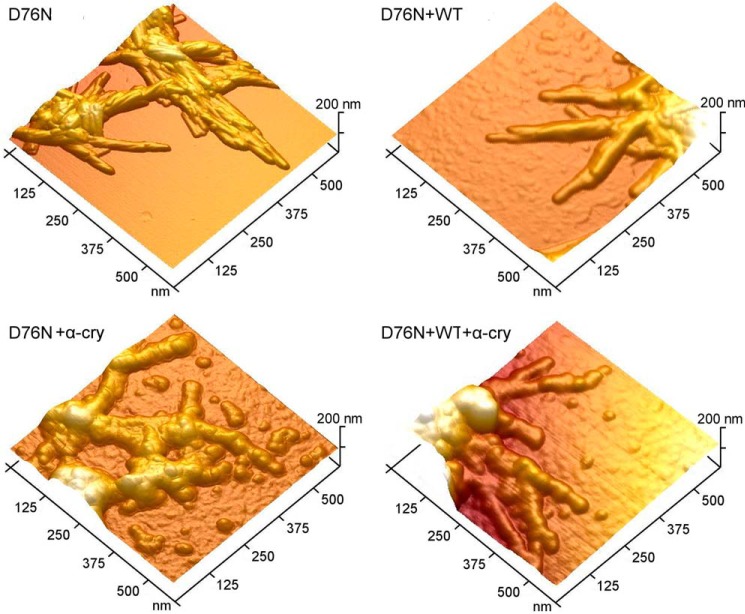
**AFM analysis of fibrils and interaction with α-crystallin.** Surface plots of topographic AFM images showing fibrillar aggregates formed by D76N β_2_m alone and by the equimolar mixture of WT and D76N β_2_m, in the absence (*top*) and presence (*bottom*) of α-crystallin (α-*cry*). Globular structures can be also observed in the *upper corner* of the image of fibrils by D76N+α-cry or in the background of the image of fibrils by D76N+WT+α-cry.

AFM was employed to visualize the interaction between α-crystallin. Fig. 12 shows representative surface plots from topographic AFM images obtained in different conditions. In the absence of the chaperone, D76N β_2_m formed bundles of straight fibrils, with smooth edges and relatively uniform diameter. The fibril height was 8.0 ± 0.3 nm and the fibril width (corrected for tip size effects, see “Experimental Procedures”) was 27 ± 1 nm. Mixing D76N and WT β_2_m resulted in fibrils of slightly larger size (height 8.9 ± 0.4 nm, width 32 ± 1 nm) but similar morphology. In the presence of α-crystallin, D76N β_2_m self-assembled into fibrils with irregular, beaded morphology and increased size (height 13.8 ± 0.5 nm, width 44 ± 2 nm) as compared with D76N β_2_m alone. In the same conditions, the size of fibrils obtained from equimolar mixtures of WT and D76N β_2_m was very similar (height 8.1 ± 0.3 nm, width 30 ± 1 nm) to that measured in the absence of the chaperone, but the fibrils displayed the same altered morphology found for D76N β_2_m in the presence of α-crystallin. For both D76N β_2_m and D76N/WT β_2_m mixtures, the fibril features indicate that α-crystallin was associated to the aggregates, in agreement with the results of gel electrophoresis experiments. Not only did the fibrils exhibit a beaded morphology, suggesting a different packing of the protein units within the fibril, but spheroidal structures were also embedded in the fibril body. In particular, the fibril ends often terminated with a globular structure, a feature that was completely absent in the fibrils formed without the chaperone. Some of these globular structures could also be observed as isolated entities in the proximity of fibrils (Fig. 12). The volume of these globular structures was roughly estimated from their measured height and width and equal to 2 × 10^3^ nm. This value is compatible with the mean volume expected for α-crystallin (1.8 × 10^3^ nm^3^), which forms hybrid oligomeric species of average mass of ∼800 kDa and 150 Å in diameter (34).

## Discussion

**FIGURE 13.**
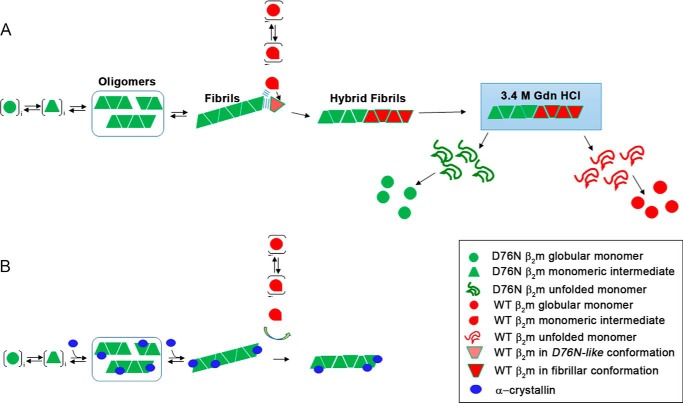
**Schematic representation of the mechanism of copolymerization of D76N and WT β_2_m.** *A,* nucleation phase only involves native globular D76N β_2_m. When D76N fibrils are formed, the WT protein can start the fibrils elongation. Surface of the edge of fibrils facilitates the fibrillary conversion of monomeric WT β_2_m. Disassembly of hybrid WT/D76N β_2_m fibrils by chemical denaturation occurs via simultaneous release of WT and variant. *B*, crystallin absorbed on D76N β_2_m fibrils prevents their seeding effect on wild-type β_2_m, which, at this state, cannot contribute to fibril elongation.

Amyloid deposition *in vitro* and *in vivo* is substantially regulated by two phases: nucleation and elongation. In systemic amyloidosis the nucleation originates from a partially folded intermediate state whose concentration can be enhanced by appropriate chemical physical conditions. At very low pH, globular proteins like lysozyme, transthyretin, and β_2_m can visit this partially folded state and self-aggregate into amyloid nuclei. Elongation of fibrils also requires a conformational transition of the monomeric protein precursor but the concentration of such an intermediate is less crucial, because the polymeric state is thermodynamically favored (33) as confirmed by the comparative analysis of the thermodynamic parameters of fibrils *versus* β_2_m precursors (Table 1). The terminal end of fibril offers a proper template to absorb the monomers, but can also play an “isomerase-like” activity in which the surface may catalyze the conversion of native-like monomers into a proper conformation suitable for fibril elongation (35). Understanding whether the mechanism of amyloid propagation of WT β_2_m occurs through a *prion-like* process (36) or secondary nucleation-dependent growth (6) is crucial to explain both the kinetics of fibrils growth *in vitro* and *in vivo* and the clinical history of systemic amyloidosis. Furthermore, this information will contribute to understand the effect of several innovative therapies including modulation of concentrations of the amyloidogenic precursor (37), stabilization of the native state to avoid the amyloid conversion (38), or a more direct process of antibody-mediated amyloid degradation (39). The discovery of the naturally amyloidogenic variant of β_2_m and the extensive characterization of the basis of amyloidogenicity of the WT protein (40) make this protein a unique model to understand whether a *prion-like* mechanism or a nucleation-dependent elongation prevails in amyloid propagation in systemic amyloidosis. Our data demonstrate that fibrillar but not the native β_2_m variant can induce the amyloid conversion of the WT β_2_m through the model described in Fig. 13. WT β_2_m delays aggregation of the D76N variant suggesting that the interaction of the two proteins in their native and/or native-like conformation does not evolve toward the formation of amyloid nuclei. The FTIR analyses indicate that, at the beginning of incubation, the structural conversion from the native protein into amyloid fibrils only involves the D76N variant, whereas the WT β_2_m remains soluble and preserves its native secondary structures. The WT protein starts its amyloid transition only when the fibrillar conversion of D76N β_2_m is complete, suggesting that copolymerization proceeds via elongation of D76N fibrils rather than a bimolecular process as proposed for the ΔN6 β_2_m variant (41). Our results on D76N β_2_m suggest a model of assembly of the hybrid fibrils in which part of the fibril is formed by variant and the other by WT β_2_m as a consequence of elongation of fibrillar seeds (Fig. 13). It is worth noting that D76N β_2_m fibrils, grown either in physiological conditions or in the presence of 20% TFE, are as stable as hybrid WT/D76N fibrils and, under conditions of dissociation the two β_2_m species are simultaneously released from the hybrid fibrils. Nevertheless, homogeneous WT fibrils obtained in 20% TFE are less stable than any other type of fibrils investigated here (Table 1) suggesting that structural rearrangement and compactness may be different if the WT protein elongates D76N β_2_m fibrils or whether it grows on its own nuclei. The FTIR analyses indicated that the secondary structures of the D76N β_2_m fibrils formed during the initial phases in the incubation of the mixture are indistinguishable from those formed by D76N β_2_m alone. These data highlight the active role of the fibrils edges in priming conformational changes of monomeric precursors and reveal that the final conformation of D76N and WT β_2_m is similar once cemented into the fibrillar structure (Fig. 13).

In such a scenario the chaperone crystallin, which is known to bind amyloid fibrils (42, 43), although unable to stop the fibrillogenesis of D76N β_2_m, can interfere with the elongation of the wild-type. After the formation of D76N β_2_m fibrils, crystallin is completely absorbed on the insoluble fibrils and, at this state, it can still play its inhibitory effect on the wild-type β_2_m elongation (Fig. 13). Therefore, we hypothesize that crystallin absorbed on fibrils may inhibit the catalytic process played by the fibrillar surface.

Overall these data underline the importance of elucidating the structure of amyloid fibrils at high resolution and with particular regard to the surface where elongation occurs. This will help to interpret the surface catalytic activity of fibrils at the molecular level and explain the mechanism of interference by chaperone and other fibrils ligands of pharmaceutical interest.

## Author Contributions

The study was conceived, designed, supervised by V. B. and S. R.; A. N., P. P. M., S. G., R. P., L. M., I. Z., A. R., D. A., and G. F. performed research. M. V., M. S., and S. M. D. contributed to experimental design and discussion. All authors analyzed and interpreted the data. The paper was written by A. N., V. B., and S. R., and reviewed and approved by all co-authors.

## Footnotes

2 The abbreviations used are:

β_2_m: β_2_-microglobulin
TTR: transthyretin
ΔN6 β_2_m: truncated wild-type β_2_m lacking the first 6 N-terminal residues
TFE: trifluoroethanol
ThT: thioflavin T
GdnHCl: guanidine hydrochloride
Δ*G*_el_: free energy of elongation
[*D*]_50%_: midpoint denaturant concentration
EM: electron microscopy
AFM: atomic force microscopy
Δ*G*_0_(H_2_O): free energy of unfolding in the absence of denaturant.
